# Extract2Chip—Bypassing Protein Purification in Drug Discovery Using Surface Plasmon Resonance

**DOI:** 10.3390/bios13100913

**Published:** 2023-10-05

**Authors:** Ana C. F. Paiva, Ana R. Lemos, Philipp Busse, Madalena T. Martins, Diana O. Silva, Micael C. Freitas, Sandra P. Santos, Filipe Freire, Evelyne J. Barrey, Xavier Manival, Lisa Koetzner, Timo Heinrich, Ansgar Wegener, Ulrich Grädler, Tiago M. Bandeiras, Daniel Schwarz, Pedro M. F. Sousa

**Affiliations:** 1 iBET, Instituto de Biologia Experimental e Tecnológica, Apartado 12, 2781-901 Oeiras, Portugal; cpaiva@ibet.pt (A.C.F.P.); ar.lemos@ibet.pt (A.R.L.); philipp.busse@ibet.pt (P.B.); m.tropamartins@lumicks.com (M.T.M.); dsilva@ibet.pt (D.O.S.); mfreitas@ibet.pt (M.C.F.); sandrasantos@ibet.pt (S.P.S.); f.freire@ibet.pt (F.F.); tiago.bandeiras@ibet.pt (T.M.B.); 2 Instituto de Tecnologia Química e Biológica António Xavier, Universidade Nova de Lisboa, Av. da República, 2780-157 Oeiras, Portugal; 3 Merck Healthcare KGaA, Frankfurter Strasse 250, 64293 Darmstadt, Germany; evelyne.barrey@astrazeneca.com (E.J.B.); lisa.koetzner@merckgroup.com (L.K.); timo.heinrich@merckgroup.com (T.H.); ansgar.wegener@merckgroup.com (A.W.); ulrich.graedler@merckgroup.com (U.G.); 4 IMoPA, CNRS, Université de Lorraine, F-54000 Nancy, France; xavier.manival@univ-lorraine.fr

**Keywords:** surface plasmon resonance, Extract2Chip, drug discovery, protein biotinylation

## Abstract

Modern drug discovery relies on combinatorial screening campaigns to find drug molecules targeting specific disease-associated proteins. The success of such campaigns often relies on functional and structural information of the selected therapeutic target, only achievable once its purification is mastered. With the aim of bypassing the protein purification process to gain insights on the druggability, ligand binding, and/or characterization of protein–protein interactions, herein, we describe the Extract2Chip method. This approach builds on the immobilization of site-specific biotinylated proteins of interest, directly from cellular extracts, on avidin-coated sensor chips to allow for the characterization of molecular interactions via surface plasmon resonance (SPR). The developed method was initially validated using Cyclophilin D (CypD) and subsequently applied to other drug discovery projects in which the targets of interest were difficult to express, purify, and crystallize. Extract2Chip was successfully applied to the characterization of Yes-associated protein (YAP): Transcriptional enhancer factor TEF (TEAD1) protein–protein interaction inhibitors, in the validation of a ternary complex assembly composed of Dyskerin pseudouridine synthase 1 (DKC1) and RuvBL1/RuvBL2, and in the establishment of a fast-screening platform to select the most suitable NUAK family SNF1-like kinase 2 (NUAK2) surrogate for binding and structural studies. The described method paves the way for a potential revival of the many drug discovery campaigns that have failed to deliver due to the lack of suitable and sufficient protein supply.

## 1. Introduction

The functional and structural characterization of drug targets and molecules is indispensable for the discovery and development of new drugs. Several experimental methods can be employed to assess the interaction between target proteins and ligands, which often require high-quality reagents—pure, pharmacologically active, and stable. Protein purification methods are widely used in drug discovery programs to supply appropriate reagents that allow for the gathering of structural and functional insights into drug targets. It combines a series of upstream and downstream techniques, from molecular biology to the recombinant protein production in different cellular systems (e.g., bacterial, insect, or mammalian) and subsequently to the separation and isolation that lead, finally, to a “pure” and pharmacologically relevant protein. This protein would ideally be stable in an optimal buffer formulation for the course of the experimental procedure [1]. Ideally, drug development programs would benefit from shortcutting this often difficult and costly task.

Due to the relevance of in-depth drug target activity assessment, downstream processing has been improved to allow for protein separation in more predictable patterns, with lower effort and higher efficiency. This has been achieved via protein construct engineering with variable affinity and solubility tag systems, coupled with innovative purification matrices for efficient and milder chromatography. In parallel, a variety of orthogonal biophysical and biochemical methods have been developed to study the molecular interactions between purified proteins and small molecules or potential protein partners. Among these methods, thermal-shift assay (TSA), nuclear magnetic resonance (NMR), isothermal titration calorimetry (ITC), fluorescence polarization (FP), and fluorescence resonance energy transfer (FRET), have been applied in drug discovery [2,3,4,5]. Surface plasmon resonance (SPR) is currently recognized as an essential technology for lead discovery and optimization. It requires the immobilization of a pure and homogeneous protein (ligand) to a sensor-chip surface and the injection of increasing concentrations of the analyte for an accurate determination of the interaction affinity and kinetic parameters [6]. Nevertheless, there are still proteins for which the purification remains a challenge, either due to low expression yield, aggregation, or degradation during or after purification, leading to an increase in production costs and prolonged timelines. In addition, current purification strategies bear the risk of removing important protein co-factors or scaffolding partners required for protein integrity or activity, ultimately hampering the development of new molecules for potentially attractive drug targets [7,8,9]. A technology that would integrate sensor-chip surface immobilization with an effortless protein purification approach could be a game-changer for targets which are difficult to study.

Protein biotinylation is a well-documented methodology that relies on the biologically strong biotin/avidin interaction affinity (KD ~10–15 M), which has already proven to be useful in a variety of applications, including the selective extraction of proteins from the expression medium for purification purposes and subsequent characterization with small molecules or potential protein partners by SPR [10,11,12]. The biotinylation process can occur through chemical or enzymatic techniques. Chemical biotinylation of a protein lacks target selectivity by modifying a broad range of similar chemical groups, while enzymatic biotinylation is highly specific and is applied in a number of applications through the action of an *Escherichia coli* (*E.coli*) biotin ligase protein termed BirA. This protein covalently attaches biotin to a lysine-residue side-chain embedded in a synthetic substrate: AviTag^TM^, a short genetically engineered fusion tag with 15 amino acids, which can be fused at either terminus of a protein [13]. A major advantage is that this enzymatic reaction can be performed either in vitro or directly in the native cellular environment [14,15].

The function and activity of a biotinylated pure protein can then be studied with high specificity and confidence by biophysical techniques such as SPR. The development of different avidin-like surface biosensors (streptavidin or neutravidin coated surface chips) and innovative immobilization strategies (His-tagged streptavidin, switchavidin) that made surface regeneration possible have recently enabled the study of interactions with irreversible compounds or inherently unstable targets [16,17]. Furthermore, the immobilization of biotinylated targets has been shown to promote an increase in surface homogeneity, binding capacity, and cycle-to-cycle reproducibility, crucial parameters for a robust kinetic characterization by SPR [17].

**Figure 1:**
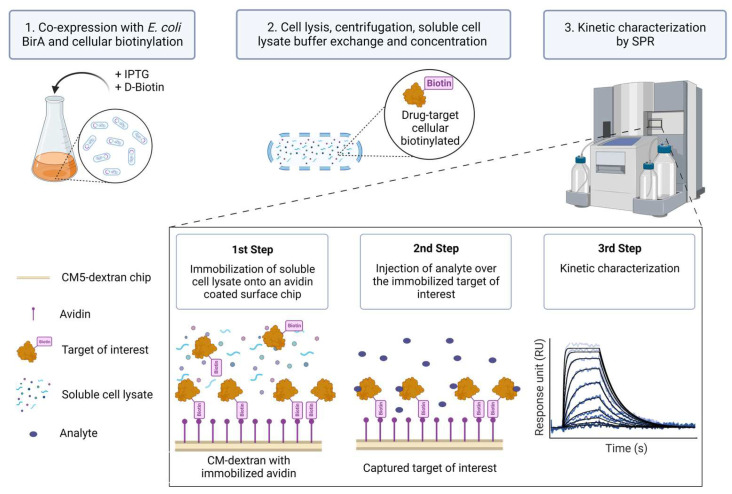
Extract2Chip method description. The target of interest is fused with an AviTag^TM^ and co-expressed with *Escherichia coli* (*E. coli*) BirA protein in the presence of D-Biotin to biotinylate the AviTag^TM^ (1). Afterward, the cells are lysed and centrifuged to separate the cellular debris, and the soluble proteins are buffer-exchanged to remove the free D-Biotin and concentrated (2). The soluble lysed material is then directly immobilized onto an avidin-coated surface chip via the biotinylated AviTag^TM^, and a series of increased concentrations of analyte (e.g., small molecules or protein partners) is injected over the immobilized target of interest in order to kinetically characterize their interaction (3). Created with BioRender.com.

Despite the aforementioned advances in protein purification and immobilization strategies, SPR kinetic studies involving biotinylated protein targets have so far, to our knowledge, only been pursued for those known to be pure and relatively stable in solution. In this report, we describe a method (Extract2Chip) that bypasses the need for protein purification, relying on cellular protein biotinylation and direct immobilization of cleared cellular content on avidin-coated sensor chip surfaces. This method consists of the co-expression of the Avi-tagged target of interest with BirA in the presence of D-biotin. The cell lysate is then buffer-exchanged to ensure free D-biotin removal, concentrated, and directly immobilized on a covalently bound avidin-coated chip via the biotinylated AviTag^TM^, without the need for surface regeneration. A series of increased concentrations of the analyte are then injected over the immobilized surface, and the kinetic and affinity parameters of the interaction are determined (Figure 1). The advantage of this method is that it provides a fast assessment and validation of the binding affinity and kinetic characterization between recalcitrant drug targets and lead molecules or protein partners via SPR.

The method was applied on four different proteins to demonstrate the versatility of the applications, as described in Table 1.

Herein, we demonstrate the broad applicability of the developed methodology and how it can be used to tackle the druggability of previously uncharacterized drug targets. We coupled cellular biotinylation to direct SPR immobilization and kinetic characterization, sparing the hurdles of highly complex purification processes.

## 2. Materials and Methods

### 2.1. Extract2Chip Method Validation—Proof of Concept 

#### 2.1.1. DNA Constructs

The coding sequence for BirA-His_6_ cloned into pET-21a(+) was purchased from Addgene. The coding sequences for BirA-FLAG and His_6_-TEV-CypD(43-207)-AviTag were synthesized and cloned into pRSF-Duet^TM^-1 in Multiple Cloning Sites (MCS) 1 and 2, respectively, by GenScript (Piscataway, NJ, USA).

#### 2.1.2. Expression and Purification of CypD and BirA

The DNA plasmids containing the His_6_-TEV-CypD(43-207)-AviTag and BirA-His_6_ and coding sequences were transformed in BL21 Star™ (DE3) pRARE2 and BL21(DE3), respectively, and grown overnight at 37 °C in LB agar plates with the respective antibiotics as selection agents. Fresh colonies were picked and grown overnight at 37 °C and 150 rpm in PB media supplemented with the respective antibiotics. Each overnight culture was diluted in PB media supplemented with the respective antibiotics to a final optical density (OD) of 0.1, then grown at 37 °C and 150 rpm until reaching an OD between 1.6 and 2.0. The proteins were expressed using 100 µM and 500 µM IPTG, respectively, overnight at 18 °C (CypD) or for 3 h at 30 °C (BirA). Finally, the cells were harvested by centrifugation at 7030× *g* and 4 °C for 15 min, then stored at −80 °C.

The cells expressing His_6_-TEV-CypD(43-207)-AviTag protein were resuspended in lysis buffer A (30 mM HEPES pH 7.5, 500 mM NaCl, 5% glycerol, 2 mM MgCl_2_, 10 mM imidazole, 1 mM TCEP, 5 U/mL benzonase and protease inhibitor cocktail (PIC) without EDTA) and disrupted in a high-pressure homogenizer (EmulsiFlex-C5, AVESTIN, Inc., Ottawa, ON, Canada) at 900 bar. The lysate was cleared by centrifugation at 30,000× *g* and 4 °C for 1 h with a JA-14 rotor, and then loaded onto a HisTrap^TM^ HP (Cytiva, Uppsala, Sweden) column equilibrated in buffer B (30 mM HEPES pH 7.5, 500 mM NaCl, 5% glycerol, 10 mM imidazole and 1 mM TCEP). The bound His_6_-TEV-CypD(43-207)-AviTag protein was eluted with a 60–140 mM imidazole gradient using buffers B and C (30 mM HEPES pH 7.5, 150 mM NaCl, 5% glycerol, 1 M imidazole and 1 mM TCEP). The fractions containing the protein of interest were injected onto a HiPrep 26/10 Desalting (Cytiva, Uppsala, Sweden) column previously equilibrated in buffer D (50 mM Tris-HCl pH 8.0, 100 mM NaCl, 5% glycerol, 1 mM EDTA and 1 mM DTT). The eluted sample was incubated with 200 U/mg TEV protease overnight at 30 °C and, afterwards, was injected onto a HisTrap^TM^ HP column equilibrated in buffer B. The flow containing the cleaved CypD(43-207)-AviTag protein was concentrated and injected onto a HiLoad 26/60 Superdex 75 Prep Grade (Cytiva, Uppsala, Sweden) column equilibrated in buffer E (30 mM HEPES pH 7.5, 150 mM NaCl, 5% glycerol, and 0.5 mM TCEP).

The cells containing the BirA-His_6_ protein were resuspended in lysis buffer F (25 mM Tris-HCl pH 8.0, 200 mM NaCl, 2 mM MgCl_2_, 10 mM imidazole, 5 U/mL Benzonase, and PIC without EDTA) and disrupted. The lysate was cleared and then loaded onto a HisTrap^TM^ HP column equilibrated in buffer G (25 mM Tris-HCl pH 8.0, 200 mM NaCl and 10 mM imidazole). The column was washed with buffer H (25 mM Tris-HCl pH 8.0, 500 mM NaCl and 10 mM imidazole), and the bound protein was eluted with a 90–190 mM imidazole gradient using buffers G and I (25 mM Tris-HCl pH 8.0, 200 mM NaCl and 1 M imidazole). The fractions with BirA-His_6_ were collected, diluted 10 times, and then injected onto a Resource^TM^ Q (Cytiva, Uppsala, Sweden) column equilibrated in buffer J (25 mM Tris-HCl pH 8.2). The protein was eluted with a 40–100 mM NaCl gradient using buffers J and K (25 mM Tris-HCl pH 8.2 and 1 M NaCl). The fractions containing the BirA-His_6_ protein were pooled and concentrated to be injected onto a HiLoad 26/60 Superdex 75 Prep Grade column equilibrated in buffer L (25 mM Tris-HCl pH 8.2 and 150 mM NaCl).

All purification steps were performed at 4 °C, and, after size exclusion chromatography, the fractions corresponding to the monomeric form of the protein were collected, concentrated, and stored at −80 °C.

#### 2.1.3. In Vitro Biotinylation of CypD Protein

To biotinylate the CypD protein in vitro, 40 µM CypD(43-207)-AviTag were incubated with 2.5 µg BirA-His_6_ protein in the presence of 50 mM Bicine pH 8.3, 10 mM ATP, 10 mM Mg(CH_3_COO)_2_, and 50 µM D-Biotin (B4501, Sigma-Aldrich, Burlington, Massachusetts, USA) for 2 h at 30 °C (as described from Avidity, LLC, Aurora, Colorado, USA). The incubated mixture was then injected onto a PD-10 Desalting (Cytiva, Uppsala, Sweden) column equilibrated in buffer D to remove the excess of D-Biotin, and the eluate was stored at −80 °C.

#### 2.1.4. Cellular Biotinylation of CypD

The pRSF-Duet^TM^-1 vector containing the BirA-FLAG and His_6_-TEV-CypD(43-207)-AviTag coding sequences was expressed as described above. In order to ensure cellular biotinylation of His_6_-TEV-CypD(43-207)-AviTag protein, 5 µg/mL D-Biotin was added to the cells upon IPTG addition. The cells were resuspended in BugBuster^®^ Protein Extraction Reagent (Novagen) supplemented with 0.1 mg/mL Lysozyme, 5 U/mL Benzonase, 1 mM PMSF, and PIC without EDTA, then placed on ice for 20 min. The lysate was cleared by centrifugation at 13,200× *g* and 4 °C for 25 min and injected onto a PD-10 Desalting column equilibrated in buffer M (30 mM HEPES pH 7.5, 150 mM NaCl, 5% glycerol, 0.5 mM TCEP, 1 mM PMSF, and PIC without EDTA) to remove the excess of D-Biotin. 

### 2.2. Extract2Chip Applied on a Recalcitrant Protein

#### 2.2.1. DNA Construct

The coding sequence for His_6_-YAP(2-268)-AviTag was synthesized and cloned into pRSF-Duet^TM^-1 in MCS 2, replacing the His_6_-TEV-CypD(43-207)-AviTag coding sequence, by GenScript. The coding sequence for TEAD1(209-426) was synthesized and cloned as in [21]. The biotinylated YAP(61-100) peptide was purchased from Biosyntan GmbH (Berlin, Germany).

#### 2.2.2. Expression and Purification of TEAD1

Production of TEAD1(209-426) was performed as in [21].

#### 2.2.3. Cellular Biotinylation of YAP

The cellular biotinylation of the YAP(2-268) protein followed the same approach as described for CypD. YAP(2-268) was expressed in BL21 Star™ (DE3) pRARE2 in PB media and induced with 50 µM IPTG during overnight at 18 °C. The PD-10 Desalting column was equilibrated with buffer N (30 mM HEPES pH 7.5, 150 mM NaCl, 2 mM MgCl_2_, 1% glycerol, 1 mM TCEP, 1 mM PMSF and PIC without EDTA).

### 2.3. Extract2Chip Applied on a Drug Target Surrogate Validation

#### 2.3.1. DNA Construct 

The coding sequences for His_6_-TEV-MARK3(48-370)-HRV3C-AviTag and the 6 designed MARK3 mutants (Table 2) were synthesized and cloned into pRSF-Duet^TM^-1 in MCS 2, replacing the His_6_-TEV-CypD(43-207)-AviTag coding sequence, by GenScript (Piscataway, NJ, USA). GST-NUAK2(1-628) was purchased from Carna Biosciences, Inc (Kobe, Japan).

#### 2.3.2. Expression and Purification of MARK3

The cells containing the cellular biotinylated His_6_-TEV-MARK3(48-370)-HRV3C-AviTag protein were resuspended in lysis buffer O (20 mM HEPES pH 7.5, 100 mM NaCl, 15% glycerol, 1 mM MgCl_2_, 1 mM PMSF, 1 mM TCEP, 5 U/mL benzonase, and PIC without EDTA) and disrupted. The lysate was cleared and loaded onto a Strep-Tactin^®^ Superflow^®^ high-capacity cartridge (IBA Lifesciences, Göttingen, Germany) column equilibrated in buffer P (20 mM HEPES pH 7.5, 100 mM NaCl, 15% glycerol, 1 mM PMSF, and 1 mM TCEP). The bound cellular biotinylated His_6_-TEV-MARK3(48-370)-HRV3C-AviTag protein was eluted after overnight incubation with HRV3C protease on-column. The peak fractions were collected and injected onto a HisTrap^TM^ HP column equilibrated in buffer Q (20 mM HEPES pH 7.5, 100 mM NaCl, 15% glycerol, 10 mM imidazole, 1 mM TCEP). The protein was eluted with a 150–200 mM imidazole gradient using buffers Q and R (20 mM HEPES pH 7.5, 100 mM NaCl, 15% glycerol, 1 M imidazole, 1 mM TCEP). The fractions containing the His_6_-TEV-MARK3(48-370) protein were pooled and concentrated to be injected onto a HiLoad 16/60 Superdex 75 Prep Grade column equilibrated in buffer S (25 mM Na Phosphate, 100 mM NaCl, 15% glycerol, 1 mM TCEP). All purification steps were performed at 4 °C, and, after size exclusion chromatography, the fractions corresponding to the monomeric form of the protein were collected, concentrated, and stored at −80 °C.

#### 2.3.3. Cellular Biotinylation of MARK3 Kinase Domain Mutants

The cellular biotinylation of MARK3(48-370) kinase domain mutants followed the same approach as described for CypD. The 6 different mutants were expressed in Rosetta^TM^ 2 in PB media, and induced with 100 µM IPTG overnight at 18 °C. The PD-10 Desalting column was equilibrated with buffer T (20 mM HEPES pH 7.5, 100 mM NaCl, 15% glycerol, 1 mM TCEP, and 1 mM PMSF).

### 2.4. Extract2Chip Applied on Validation of Ternary Protein Complexes

#### 2.4.1. DNA Construct

The coding sequence for His_6_-DKC1(1-514)-AviTag was synthesized and cloned into pRSF-Duet^TM^-1 in MCS 2, replacing the His_6_-TEV-CypD(43-207)-AviTag coding sequence, by GenScript. The coding sequences for His_6_-RuvBL1(1-456)/RuvBL2(1-463), His_6_-RuvBL1_D302N/RuvBL2_D299N, and His_6_-RuvBL1_ΔDII/RuvBL2_ΔDII were synthesized and cloned as described in [22].

#### 2.4.2. Expression and Purification of RuvBL1/RuvBL2

The production of the His_6_-RuvBL1(1-456)/RuvBL2(1-463), His_6_-RuvBL1_D302N/ RuvBL2_ D299N, and His_6_-RuvBL1_ΔDII/RuvBL2_ΔDII constructs was performed as in [22].

#### 2.4.3. Cellular Biotinylation of DKC1

The cellular biotinylation of the DKC1(1-514) protein followed the same approach as that described for CypD. DKC1(1-514) was expressed in BL21(DE3) in LB media and induced with 500 µM IPTG overnight at 18 °C. The PD-10 Desalting column was equilibrated with buffer U (50 mM HEPES pH 6.5, 750 mM KCl, 0.5 mM EDTA, and 0.5 mM TCEP).

All soluble cell lysates were concentrated and stored at −80 °C. The cellular biotinylated proteins were identified by Western blot, using a HisTag antibody (H1029 and A2429, Sigma-Aldrich), Streptavidin AP (21324, ThermoFisher Scientific, Waltham, MA, USA) or HRP Conjugate (SNN1004, ThermoFisher Scientific, Waltham, MA, USA).

### 2.5. SPR Assays

#### 2.5.1. Characterization of CypD Interaction with Ligand CYPD-27

The purified CypD(43-207) protein was immobilized onto a CM5 Series S sensor chip (Cytiva, Uppsala, Sweden) using standard amine coupling with 20 mM HEPES pH 7.4, 150 mM NaCl, 0.1 mM EDTA, 0.3 mM DTT, and 0.05% Tween-20 as background buffer. The carboxymethylated surface of the chip was activated with 400 mM 1-ethyl-3-(3-dimethylaminopropyl)-carbodiimide (EDC) and 100 mM N-hydroxysuccinimide (NHS) for 7 min. The protein was diluted in 10 mM sodium phosphate with a pH of 7.0 to a concentration of 10 µg/mL and coupled to the surface with 5 and 10 min of injection time at a flow rate of 10 µL/min in order to reach 400 to 900 response units (RU). The remaining activated groups were blocked with 1 M ethanolamine-HCl pH 8.5 for 7 min. 

The in vitro biotinylated and the cellular biotinylated CypD(43-207) proteins were immobilized onto Series S sensor chips SA (Cytiva, Uppsala, Sweden) using standard streptavidin–biotin coupling. The surfaces of the chips had previously been washed with three injections of 1 M NaCl and 50 mM NaOH for 1 min each. The in vitro biotinylated protein was diluted to the same concentration and coupled to the surface with 1 and 4 min of injection time in order to reach 600 to 4000 RU. The soluble cell lysate containing the cellular biotinylated protein was diluted to a concentration of 100 µg/mL and coupled to an SA chip surface, with a 90 s injection time, to reach 700 RU.

The CypD known inhibitor CYPD-27 [18] (alternatively named compound 12 in [19] or compound 2 in [20]) was tested on all immobilized samples at 10 different concentrations using a 2-fold dilution series, with 2 µM as the highest tested concentration. The interaction analysis cycles consisted of a 120 to 150 s sample injection (association phase), followed by 180 s of running buffer flow (dissociation phase) at a flow rate of 30 µL/min. The running buffer was composed of 20 mM HEPES pH 7.4, 150 mM NaCl, 0.1 mM EDTA, 1 mM DTT, 0.05% Tween-20, and 2% DMSO, and the experiment was performed at 25 °C.

#### 2.5.2. Characterization of YAP:TEAD1 Interaction in Presence of Small Molecules

The cellular biotinylated YAP(2-268) protein, as well as the biotinylated YAP(61-100) peptide, were immobilized using a similar approach as that described previously. However, neutravidin protein (ThermoFisher Scientific) was used as the selected pre-coated surface on a CM5 Series S sensor chip instead of streptavidin. Hence, prior to YAP immobilization, neutravidin was diluted to 50 µg/mL in 20 mM sodium citrate with a pH of 4.5 and covalently immobilized on the CM5 chip, following activation by EDC/NHS, for 20 min to reach 10,000 RU. The soluble cell lysate containing the biotinylated YAP(2-268) and the pure biotinylated YAP(61-100) peptide were diluted to 100 µg/mL and 0.2 µg/mL in background buffer and immobilized for 200 s and 40 s, respectively, to reach 200 RU and 30 RU. The remaining activated groups were then blocked with ethanolamine-HCl. The TEAD1(209-426) protein was directly dissolved in running buffer (20 mM Tris-HCl pH 7.4, 150 mM NaCl, 5 mM MgCl_2_, 0.1 mM EGTA, 0.05% CHAPS) in the absence or presence of saturating concentrations of known binders (30 µM TED-347 [23] and peptide 17 [24]) to a concentration of 3 µM, then incubated for 18 h at 30 °C. TEAD1 apo-form or incubated with compound/peptide was then injected over the immobilized YAP(2-268) surfaces at 10 different concentrations using a 2-fold dilution series. The interaction analysis cycles consisted of 120 s of sample injection followed by 280 s of running buffer flow. The experiment was performed at 15 °C. To ensure that the surface integrity of the cellular biotinylated YAP remained intact and that the interaction kinetic profile with TEAD1 was restored, an additional injection of TEAD1 apo-form was performed at the end of each experiment.

#### 2.5.3. Characterization of NUAK2 Surrogate (MARK3 WT and Mutants) with GSK461364A

The purified GST-NUAK2(1-628) was immobilized onto a NTA sensor chip (Cytiva, Uppsala, Sweden) via amine coupling with 20 mM HEPES pH 7.4, 100 mM NaCl, and 0.05% Tween-20 as background buffer. The carboxymethylated surface of the chip was activated, and the protein was immobilized for 5 and 10 min at 20 µg/mL in order to reach 2500 to 3800 RU. The remaining activated groups were then blocked with ethanolamine-HCl.

The purified His_6_-TEV-MARK3(48-370) was immobilized using the same approach as GST-NUAK2(1-628). The protein was diluted to 3 µg/mL and coupled to the surface with 5 and 12 min of injection time to reach 1000 to 1700 RU.

The cellular biotinylated MARK3 NUAK2-like mutants were immobilized using the approach described in Section 2.5.2. The soluble cell lysates were diluted to a final concentration of 200 µg/mL, then injected over the neutravidin surfaces for 200 or 1400 s in order to reach 1000 to 2300 RU.

The NUAK2 inhibitor GSK461364A [25] was injected into all immobilized samples at 10 different concentrations using a 2-fold dilution series, with the highest tested concentration reaching 0.5 µM for the GST-NUAK2(1-628) protein and His_6_-TEV-MARK3(48-370) mutants and 30 µM for the His_6_-TEV-MARK3(48-370) WT protein. The interaction analysis cycles consisted of 140 s of sample injection followed by 350 to 600 s of running buffer flow. The running buffer was composed of 20 mM HEPES pH 7.4, 100 mM NaCl, 5 mM MgCl_2_, 1 mM EGTA, 1 mM DTT, 0.05% Tween-20, and 2% DMSO, and the experiment was performed at 15 °C.

#### 2.5.4. Characterization of DCK1:RuvBL1/RuvBL2 Interaction

The cellular biotinylated His_6_-DKC1(1-514)-AviTag was immobilized using the approach described in Section 2.5.2. The soluble cell lysate was diluted to 15 µg/mL and coupled with 150 and 300 s injection times to reach 130 to 175 RU. His_6_-DKC1(1-514)-AviTag was directly diluted in the sample buffer (50 mM HEPES pH 6.5, 750 mM KCl, 0.5 mM EDTA and 0.5 mM TCEP), which also served as a background buffer during the capture. 

His_6_-RuvBL1(1-456)/RuvBL2(1-463), His_6_-RuvBL1_D302N/RuvBL2_D299N and His_6_-RuvBL1_ΔDII/ RuvBL2_ΔDII were then injected over the immobilized DKC1 surfaces at 10 different concentrations using a 2-fold dilution series, at the highest concentration of 0.05 µM. The running buffer used during the experiment contained 20 mM NaKPi pH 7.5, 150 mM NaCl, 5 mM MgCl_2_, 1 mM DTT, and 0.05% Tween^®^ 20, as previously reported [26]. The interaction analysis cycles consisted of 220 s of sample injection followed by 600 s of running buffer at 25 °C.

All sensorgrams were processed by first subtracting the binding response recorded from the control surface (reference spot, where only the avidin protein is immobilized), followed by subtraction of the buffer blank injection from the reaction spot. All datasets were fitted to a simple 1:1 Langmuir interaction model to determine the kinetic rate constants, and the interaction affinity was additionally calculated at a steady state when applicable. In cases where small molecules were present during the assay, a DMSO solvent correction (1–3%) was applied to account for variations in bulk signal and to achieve high-quality data. The experiments with immobilized purified proteins were performed on a Biacore 4000 (Cytiva, Uppsala, Sweden), and the interaction was evaluated using the provided Biacore 4000 evaluation software, while the experiments with soluble cell lysates on a Biacore T200 (Cytiva, Uppsala, Sweden) used the provided Biacore T200 evaluation software. All SPR experiments were performed in duplicate.

## 3. Results and Discussion

### 3.1. Extract2Chip Method Validation

#### The Study of CypD:Inhibitor Interaction Kinetics

Cyclophilin D (CypD) is a mitochondrial matrix protein with cis-trans peptidyl prolyl isomerase (PPIase) activity. It is involved in the formation and regulation of the mitochondrial permeability transition pore, which leads to the loss of mitochondrial membrane potential, mitochondrial swelling, rupture of the outer membrane, and necrotic cell death [27,28]. CypD has been considered a potential therapeutic drug target for several diseases that involve mitochondrial dysfunction, oxidative stress, or cell necrosis, such as ischemia-reperfusion injury of the heart and the brain, muscular dystrophies, and cancer [29,30]. 

**Figure 2:**
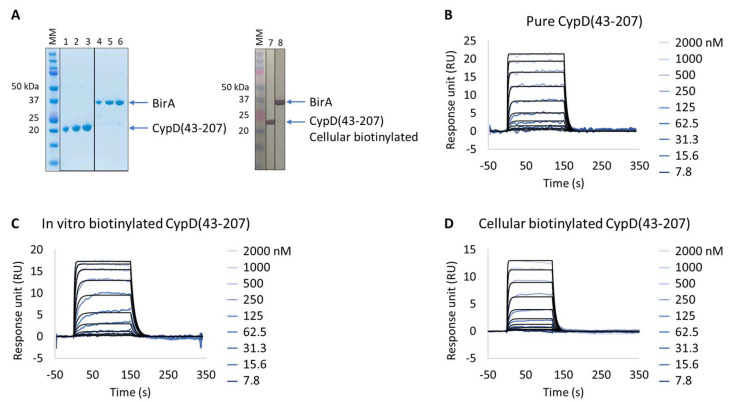
Extract2Chip method validation on Cyclophilin D (CypD). SPR kinetic characterization of CypD’s interaction with a known inhibitor (CYPD-27). (**A**) SDS-PAGE analysis of pure CypD(43-207) and BirA proteins (left) and confirmation of CypD(43-207) cellular biotinylation with BirA by western blot (right). MM, molecular marker. Lanes 1–3: 1, 2, and 3 µg of pure CypD(43-207). Lanes 5–6: 1, 2, and 3 µg of pure BirA. Lanes 7 and 8: soluble cell lysates showing the detection of CypD(43-207) and BirA using anti His-Tag or Flag-Tag antibodies, respectively. (**B**–**D**) Kinetic characterization of pure (**B**), in vitro biotinylated (**C**), or cellular biotinylated (**D**) CypD(43-207) interaction with CYPD-27. The sensorgrams show the interaction responses of different immobilized CypD(43-207) proteins upon injection of CYPD-27 (t = 0 s) at increasing concentrations or upon washing (t = 120 (**D**) or 150 s (**B**,**C**)). X-axis: time (s); Y-axis: response unit (RU).

The human CypD protein is a well-characterized drug target whose expression and purification procedures, as well as SPR-based binding assay, are well described, and for which several small-molecule inhibitors have already been developed [20], making this protein a suitable model for validation of the Extract2Chip method. For this purpose, CypD was kinetically characterized with a reference compound via a direct comparison approach using three different preparation formats: (i) as purified (Figure 2A,B); (ii) in vitro biotinylated (Figure 2C); or (iii) cellular biotinylated (Figure 2A,D).

The expression of all proteins had previously been validated by SDS-PAGE and identified by Western blot against FLAG-tagged BirA and biotinylated target protein. The characterization of the purified CypD interaction with the small molecule showed resolved binding kinetics with a calculated binding affinity of 2.1 × 10^−7^ ± 1.5 × 10^−8^ M, in agreement with previously reported data [20]. The results further demonstrate that both the in vitro and cellular biotinylated CypD extracts are capable of straightforward immobilization on SPR streptavidin-coated chips and share identical interaction affinities and kinetics compared to the purified protein (Table 3 and Figure S1). It is important to emphasize that, regardless of the heterogeneity of the injected cellular extract lysates, it was possible to obtain a homogeneous surface without any detected surface drift during the experiment, a key feature for the accurate determination of the interaction kinetic parameters in SPR assays.

The Extract2Chip approach was also applied to three other drug targets previously known to be recalcitrant, and, consequently, from in-depth biophysical characterizations excluding proteins, to elucidate whether this method could be applicable to unstable proteins or a broader spectrum of applications: identification of protein–protein interaction inhibitors; screening for optimal site-directed protein surrogate mutants to enable structural studies; and confirming the interaction between heterologously expressed components of cellular super-complexes.

### 3.2. Extract2Chip Applied on a Recalcitrant Drug-Target

#### Evaluation of YAP:TEAD1 Interaction Inhibition with Small Molecules

The Hippo signaling pathway is a well-conserved pathway that regulates cell proliferation, survival, differentiation, fate determination, organ size, and tissue homeostasis [31]. The transcriptional enhanced associated domain (TEAD) protein family contains four paralogous transcription factors (TEAD1, TEAD2, TEAD3, and TEAD4) that control gene expression in response to the Hippo signaling pathway. These are activated upon binding to transcriptional coactivators, such as Yes-associated protein (YAP), transcriptional co-activator with PDZ-binding motif (TAZ), and vestigial-like (VgLL) and p160 proteins, with YAP being one of the main coactivators [32]. Previous studies have recognized the YAP:TEAD complex as a drug target in cancer, since the deregulation of the Hippo signaling pathway and the overexpression of the two proteins have been associated with different types of cancers, with key druggable regions within TEAD (P-pocket) and in the complex interface already identified [31,32].

**Figure 3:**
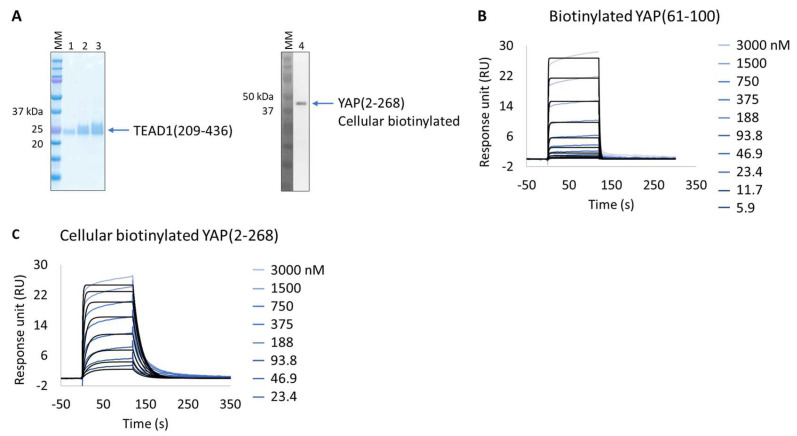
Extract2Chip applied on a recalcitrant protein. SPR kinetic characterization of transcriptional enhancer factor TEF (TEAD1) with synthetic biotinylated Yes-associated protein (YAP) peptide and cellular biotinylated YAP protein. (**A**) SDS-PAGE analysis of pure TEAD1(209-436) protein (left) and confirmation of YAP(2-268) cellular biotinylation by Western blot (right). MM, molecular marker. Lanes 1–3: 1, 2, and 3 µg of pure TEAD1(209-436). Lane 4: soluble cell lysates showing the detection of YAP(2-268) using His-Tag antibody. (**B**,**C**) Kinetic characterization of immobilized pure biotinylated YAP(61-100) (**B**) and cellular biotinylated YAP(2-268) (**C**) with TEAD1(209-436). The sensorgrams show YAP proteins’ responses upon injection of TEAD1 (t = 0 s) at increasing concentrations or upon washing (t = 120 s). X-axis: time (s); Y-axis: response unit (RU).

The in vitro characterization of YAP:TEAD interaction is an important tool for the validation of potent protein–protein interaction inhibitors. YAP is an intrinsically disordered protein, only a small segment of which is proposed to be directly involved in TEAD binding, YAP(61-100). This protein wraps around the globular surface of TEAD, forming three highly conserved interfaces (YAP residues 52-58, 61-73, and 86-100) [33]. The purification of two extended versions of this construct (YAP residues 50-171 and 2-268) results in an unstable protein with significant degradation and low production yield (50 µg/L of culture); hence, it is difficult to isolate and characterize. Alternatively, and bypassing its purification, we applied the Extract2Chip method to characterize the interaction of cellular immobilized biotinylated YAP(2-268) with pure TEAD1(209-426) and compared it with the shorter synthetic biotinylated YAP(61-100) peptide (Figure 3A–C). The results show that the cellular biotinylated YAP(2-268) was successfully expressed and immobilized on neutravidin-coated surfaces and interacted with TEAD1(209-426) with a higher affinity (K_D_ = 4.5 × 10^−8^ ± 1.6 × 10^−8^ M) than that observed for the interaction with the immobilized YAP(61-100) peptide (K_D_ = 1.1 × 10^−6^ ± 3.7 × 10^−8^ M) (Table 4 and Figure S2). To our knowledge, this is the first time a significant gain in affinity has been reported using the extended version of YAP(2-268), suggesting that additional surface contacts in the YAP(2-268):TEAD1 complex interface may enhance the overall interaction affinity, in particular those recently reported to play a role in complex formation (YAP residues 52-58) [34,35].

**Figure 4:**
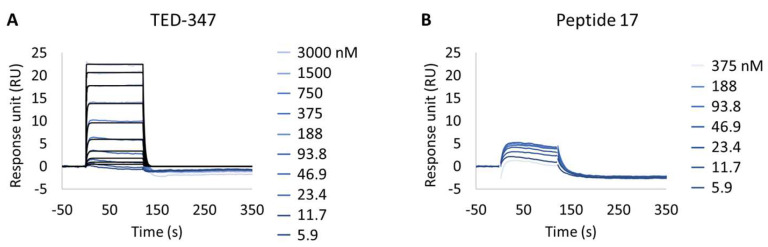
Inhibition of protein–protein interaction promoted by two different TEAD1 engagers. (**A**,**B**) Kinetic characterization of the interactions between immobilized cellular biotinylated YAP(2-268) and TEAD1(209-436) in running buffer supplemented with 30 µM TED-347 (**A**) or 30 µM Peptide 17 (**B**). The sensorgrams show the interaction responses of immobilized cellular biotinylated YAP(2-268) upon injection of increasing concentrations of TEAD1 (t = 0 s) saturated with different small molecules or upon washing (t = 120 s). X-axis: time (s); Y-axis: response unit (RU).

Following this observation, the study of potential inhibitors of the YAP:TEAD complex interaction was further investigated in the cellular biotinylated YAP(2-268) protein. Two alternative approaches are known to disturb the PPI. The closest method works via a TEAD surface binder that blocks the YAP binding domains (e.g., Peptide 17) [24]. A more sophisticated allosteric mechanism is also described, showing that occupancy of the deeply buried lipidation pocket of TEAD with an artificial ligand prevents the PPI (e.g., TED-347) [23]. The binary complex formation of TEAD1 to the different TEAD binders was determined using biochemical assays or SPR at different timescales, and it was found to be 3.4 × 10^−7^ M (IC_50_) or 1.4 × 10^−6^ M (K_D_), respectively, for the lipidation pocket binder and 8.3 × 10^−9^ M (K_D_) for Peptide 17 (data not shown). TEAD1(209-426) was incubated with saturating concentrations of each binder and injected onto biotinylated YAP(2-268) surfaces, up to a maximum concentration of 3 µM. The selected TEAD1 concentration and chosen compound to TEAD1 ratios in the solution were ensured to be 20 times above the KDs determined from binary complex formation studies, after 18 h of incubation at 30 °C. The lipid-pocket engager TED-347 displayed a decrease in affinity (Figure 4A) when compared with TEAD1 apo-form, whereas the surface binder Peptide 17 (Figure 4B) significantly suppressed the interaction between the TEAD1 and YAP proteins (Table 5 and Figure S3). This finding is in line with the observation that Peptide 17 exhibits a higher affinity for TEAD1 compared to YAP:TEAD1 interaction [36]. It is important to note that the surface integrity of YAP(2-268) was carefully monitored and confirmed to be intact throughout the entire time-course of the experiment, by re-injecting TEAD1-apo at the end of all assays and observing the same kinetic profile as in the beginning of the experiment. These results show the importance of this developed methodology in contributing to the development of new approaches for the study of intrinsically disordered proteins in drug discovery campaigns.

### 3.3. Extract2chip for Drug Target Surrogate Validation

#### Unraveling NUAK2 Optimal Surrogates Using MARK3

NUAK family kinase 2 (NUAK2) is considered one of the bona fide effector proteins downstream of YAP and has recently emerged as an alternative way of targeting the Hippo signaling pathway [37]. It belongs to the AMPK protein kinase family, acting as a critical sensor coupling cellular energy status to cell growth and proliferation [38]. The deregulation of NUAK2 has profound effects on cancer development and tumor progression, and is seen as a potential therapeutic target for several cancer-related diseases [39,40]. The NUAK2 amino acidic sequence (1-628) is predicted to bear two main disordered regions (355-493; 531-562), with the kinase domain comprising residues 53-303. To our knowledge, there is no available published crystal structure of the full-length or NUAK2 kinase domain, even taking into consideration the highly conserved fold of the kinase domain. Nevertheless, NUAK inhibitors have already been described to bind both NUAK1 and NUAK2 isoforms, presumably acting as ATP-competitive inhibitors WZ4003 and HTH-01-015 [41]. A specific NUAK2 inhibitor has yet to be described, and understanding the molecular differences between NUAK1 and NUAK2 would significantly contribute to the generation of more selective inhibitors. MARK3 is one of the closest AMPK-related kinases to NUAK2 (51% amino acid identity with respect to the kinase domain), and a crystal structure of its kinase domain is already available [42], making it the preferable NUAK2 surrogate kinase to perform site-directed protein mutant screens for structural studies enablement with NUAK2 inhibitors. In this study, six different MARK3-AviTag NUAK2-like mutants (Table 2) were designed with respect to the amino acidic residues present in the conserved kinase core, namely, at the two structurally and functionally distinct lobes that contribute in unique ways to both the catalysis and regulation of any kinase, the C- and N-lobes, respectively, in particular at the DFG motif and glycine-rich loop, including neighboring residues.

**Figure 5:**
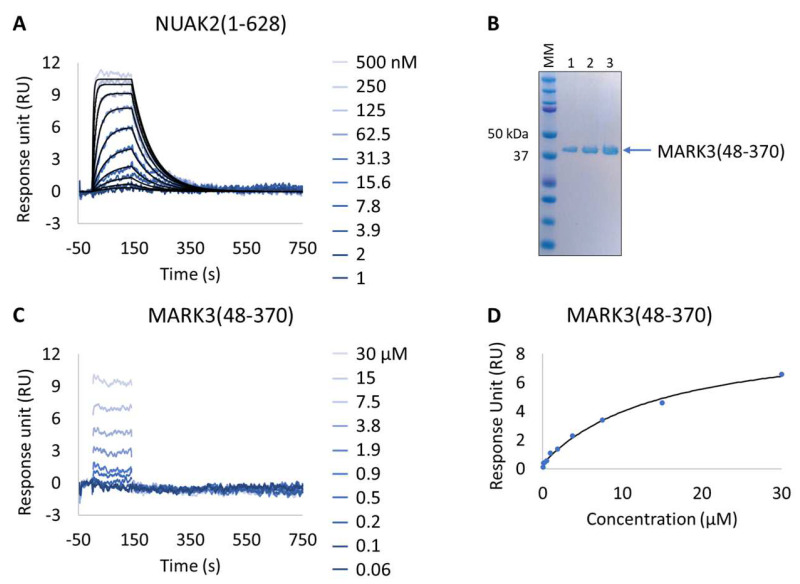
Characterization of a known NUAK family SNF1-like kinase 2 (NUAK2) inhibitor on NUAK2 and MARK3 proteins. (**A** and **C**) SPR kinetic characterization of the interaction between pure NUAK2(1-628) (**A**) and pure MARK3(48-370) (**C**) with GSK461364A. The sensorgrams show the responses of the interaction between immobilized NUAK2(1-268) (**A**) and MARK3(48-370) (**C**) after injection of increasing concentrations of GSK461364A (t = 0 s) or upon washing (t = 140 s). X-axis: time (s); Y-axis: response unit (RU). (**B**) SDS-PAGE analysis of pure MARK3(48-370) protein. MM, molecular marker. Lanes 1–3: 1, 2, and 3 µg of pure MARK3(48-370). (**D**) Steady-state affinity fit for the interaction between MARK3(48-370) and GSK461364A. X-axis: concentration of GSK461364A (µM); Y-axis: response unit (RU).

The Extract2Chip approach was applied to all MARK3 mutants for which expression was observed (mutants 2–6), thus excluding mutant 1. The characterization of MARK3 mutants’ interaction with a known PLK1 inhibitor showing NUAK1/2 inhibition in a kinase panel screen (GSK461364A) was performed. For comparison, the same inhibitor was also tested via SPR on pure NUAK2(1-628) and MARK3(48-370) (thereafter referred to as wild-type—WT) surfaces immobilized via amine coupling (Figure 5 and Table 6). As expected, a resolved binding kinetics interaction with a calculated K_D_ of 1.9 × 10^−8^ ± 4.3 × 10^−9^ M was observed for NUAK2 surfaces (Figure 5A), in contrast with the transient binding behavior detected for MARK3 WT (Figure 5C). 

**Figure 6:**
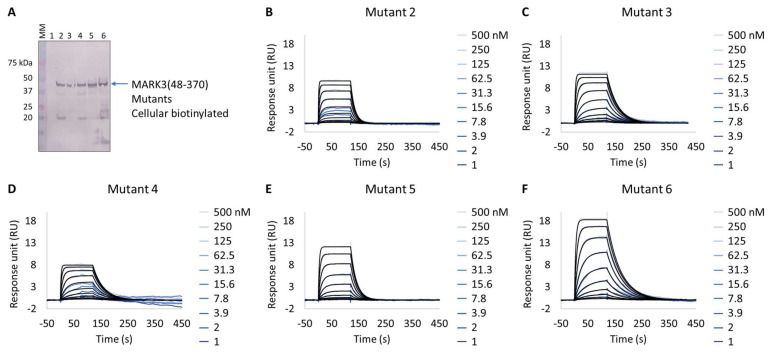
Extract2Chip applied to drug target surrogate validation. SPR kinetic characterization of the interaction between MARK3(48-370) Mutants 1–6 and NUAK2 inhibitor GSK461364A. (**A**) Western blot detection of MARK3(48-370) Mutants 1–6 in soluble cell lysates, after co-expression with BirA, using Streptavidin AP Conjugate. Lanes 1–6, MARK3(48-370) mutants 1 to 6. (**B**–**F**) Kinetic characterization between cellular biotinylated MARK3(48-370) Mutants 1–6 and GSK461364A, respectively. The sensorgrams show the responses of the interaction between different immobilized cellular biotinylated MARK3(48-370) mutants after the injection of increasing concentrations of GSK461364A (t = 0 s), or upon washing (t = 140 s). X-axis: time (s); Y-axis: response unit (RU).

Interestingly, all expressed MARK3 mutants’ interactions with GSK461364A showed resolved binding kinetics with a calculated K_D_ comparable to that of the purified NUAK2 (Figure 6B–F and Table 7). This is particularly striking for mutants 3 to 6, where additional mutations directed to MARK3 DFG motif and glycine-rich loop were designed and shown to be essential for the re-shaping of the MARK3 kinase domain with NUAK2-like interaction features. In the absence of a NUAK2 crystal structure, the co-crystal structure of a MARK3 kinase domain mutant bearing NUAK1/2 inhibitors could be useful for the design of selective NUAK2 inhibitors. In that respect, the Extract2Chip method was successfully applied as a fast screening tool for the characterization of different kinase mutants by SPR, indicating prospective advantages for similar approaches.

### 3.4. Extract2Chip for Validation of Ternary Protein Complexes

#### Exploring the DKC1:RuvBL1/RuvBL2 Cellular Complex

Cellular processes like RNA modifications or telomerase activity are mediated by snoRNPs [43]. These complexes consist of small nucleolar non-coding RNAs (snoRNAs) and protein components. The box H/ACA snoRNP and, in particular, its central core protein Dyskerin Pseudouridine Synthase 1 (DKC1)—also called Dyskerin or NAP57—are responsible for the isomerization of the RNA base uridine to pseudouridine [44]. This alteration promotes an additional hydrogen bond in the RNAs’ major groove, leading to increased stability and enhanced stacking properties [45,46]. Uridine isomerization is one of the most abundant RNA modifications, and it occurs in many types of RNA (mRNA, tRNA, rRNA, snRNA, and snoRNA) [46,47]. Hence, dysfunctional pseudouridine synthesis in mammals reduces translation rats and fidelity, and it is associated with several diseases, such as dyskeratosis congenita and numerous types of cancer [48,49]. DKC1 alone is a highly unstable protein with a well-known disordered C-terminal tail, and it is proposed to be chaperoned by SHQ1 and other protein clients during snoRNP biogenesis, protecting it from aggregation, degradation, and unspecific RNA binding [50,51]. One such client is the RT2P complex, composed of PIH1D1, RPAP3, and AAA+ ATPases RuvBL1/RuvBL2, known to directly interact with the DKC1:SHQ1 complex. In particular, RuvBL1/RuvBL2 is proposed to interact with the DKC1 C-terminal tail, effecting SHQ1 release in an apparent ATP-independent fashion [52]. 

**Figure 7:**
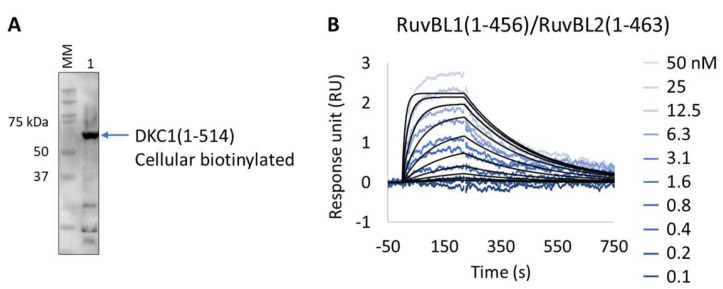
Extract2Chip applied for the validation of ternary complex formation. (**A**) Western blot identifying biotinylated Dyskerin pseudouridine synthase 1 (DKC1) (1-514) from soluble cell lysates, after co-expression with BirA, using streptavidin HRP conjugate. (**B**) SPR kinetic characterization of the interaction between immobilized cellular biotinylated DCK1(1-541) and RuvBL1(1-456)/RuvBL2(1-463). The sensorgrams show the responses of the interaction between DCK1(1-514) and RuvBL1(1-456)/RuvBL2(1-463) after injection at increasing concentrations (t = 0 s) or upon washing (t = 200 s). X-axis: time (s); Y-axis: response unit (RU).

We applied the Extract2Chip method to validate the interaction of the unstable DKC1 protein with the RuvBL1/RuvBL2 ATPases complex and to assess the impact of ATP binding on the formation of this ternary complex. The results show that the expression and immobilization of cellular biotinylated DKC1(1-514) on neutravidin-coated surfaces was achieved (Figure 7A,B), and the interaction with RuvBL1(1-456)/RuvBL2(1-463) was validated with a calculated KD of 2.3 × 10^−8^ ± 2.9 × 10^−9^ M (Table 8). Identical results were obtained in the presence of saturating concentrations of the non-hydrolysable ATPγS (NU-406, Jena Bioscience) or upon injection of the ATP hydrolysis RuvBL1/RuvBL2 mutant (RuvBL1_D302N, RuvBL2_D299N) (data not shown), confirming that ATP binding has no impact on the established interaction. Interestingly, no interaction was detected after injection of RuvBL1_ΔDII/RuvBL2_ΔDII devoid of its unique regulatory domain (Domain II, DII), suggesting that it is essential for the formation of the ternary complex. The developed methodology has been proven to be effective for studying complex, high-molecular-weight multimeric protein assemblies, particularly when precise protein immobilization with low surface density is essential to prevent avidity issues.

## 4. Conclusions

Purification is one of the major challenges of drug discovery programs. Despite all developments in recent years in downstream processes and technologies, there are yet many proteins that remain extremely difficult to obtain in pure, functional, and stable assemblies, which is essential for the development of new potential drug molecules. The Extract2Chip method aims at mitigating the difficulties associated with purifying unstable proteins, but also at enabling a fast screen of multiple biotinylated drug-target construct variants directly from biotin-cleared cell lysates, in order to support drug discovery initiatives.

The methodology consists of bypassing protein purification by using cell lysates enriched in biotinylated drug targets to kinetically characterize their interaction with small molecules or protein partners via SPR. The approach requires a pre-coating of the chip surface with an optimized density of either streptavidin or neutravidin. Alternatively, commercially available SA or NA chips (Cytiva, Uppsala, Sweden) could also be used for this purpose, but fine-tuning of captured surface densities is advised, since oversaturation could result in mass transport limitation effects or heterogeneity of the surface sites, leading to suboptimal binding kinetics.

**Figure 8:**
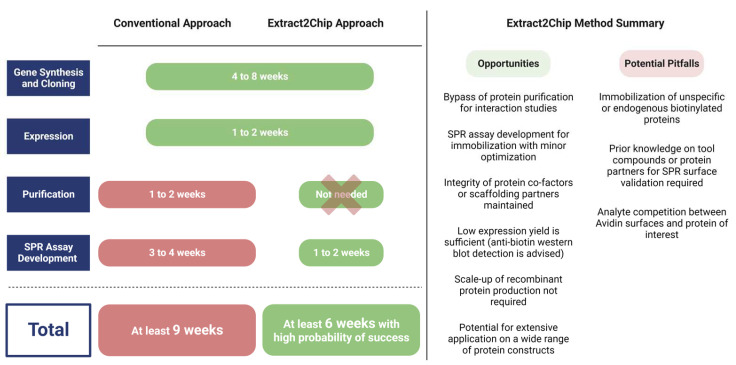
Average timespan comparison between conventional and Extract2Chip approaches, highlighting its opportunities and potential pitfalls. The Extrat2Chip method has enabled a significant reduction in the average timespan required for drug discovery projects by circumventing the downstream processes. When compared to the conventional approach, Extract2Chip can be useful for a fast and accurate assessment on the kinetic characterization between drug targets and lead molecules or protein partners, including those for which the purification failed or became unfeasible (e.g., YAP, DKC1). Created with BioRender.com.

The method was initially validated for the human CypD protein, a well-characterized drug target with previously identified high-affinity compounds, and subsequently applied to three different targets, each one representing a typical obstacle for drug discovery programs: (i) low expression levels (YAP), (ii) impaired purification (YAP, DKC1), and (iii) difficulty of crystallization (NUAK2). Extract2Chip was successfully applied for the different approaches described herein, with all binding traces following single-site pseudo-first-order binding kinetics, often only possible in the presence of highly homogeneous and stably immobilized surfaces. In addition, shorter timelines were achieved for all examples (Figure 8), from gene cloning to interaction studies, in comparison with the classical purification strategies. This is mainly due to the absence of any chromatographic step or SPR assay development, which is often required for every new purified protein construct, but is also related to the throughput of the SPR machine, which may vary considerably (from 4 to 32 independent detection spots), further increasing the number of potential protein construct variants tested per experiment. The significance of the last feature becomes particularly evident in rapid mutagenesis studies, as exemplified by the study conducted on MARK3. When coupled with integrated robotic workstations, this feature opens up the possibility of applying the methodology to a vast array of protein constructs without the requirement for protein purification, increasing the throughput for optimal protein construct selection.

Alternative sensor-chip based readout instruments, such as the bio-layer or grating couple interferometry (BLI, Creoptix WAVE), could also make use of the described method, with anticipated minor adaptations.

Finally, the Extract2Chip method could be considered as an alternative to the qualitative assessment conveyed by typical pull-down assays. It provides a more quantitative approach, with measurable interaction kinetics of potential protein–protein interactions, an essential step toward understanding the molecular basis of protein function and identifying relevant biological pathways. Future investment in understanding the Extract2Chip applicability in eukaryotic expression systems will add significant value to the method, especially considering the role that post-translational modifications often play in protein interaction studies.

## Figures and Tables

**Table 1:** Extract2Chip applications and their description and objectives.

Extract2Chip Applications	Description and Objectives
1. Proof of concept	In vitro vs. cellular biotinylation of Cyclophilin D (CypD) To gain insights on the method’s capacity to evaluate the interaction kinetic profile with a known inhibitor [18,19,20]
2. Recalcitrant proteins	Cellular biotinylation of the finicky Yes-associated protein (YAP), a transcriptional co-activator known to interact with the transcriptional enhancer factor TEF (TEAD) To investigate the inhibition of a protein–protein interaction promoted by different TEAD binders
3. Drug target surrogate validation	Robust platform to evaluate six different cellular biotinylated MAP/microtubule affinity-regulating kinase 3 (MARK3) kinase domain mutants To assess the interaction profile with a known NUAK family SNF1-like kinase 2 (NUAK2) inhibitor and identify the optimal NUAK2-like surrogate to be pursued in structural studies
4. Validation of ternary protein complexes	Cellular biotinylation of the unstable protein H/ACA ribonucleoprotein complex subunit Dyskerin pseudouridine synthase 1 (DKC1), the catalytically active protein present in box H/ACA small nucleolar ribonucleoproteins (snoRNPs) To evaluate protein–protein interactions with AAA+ ATPases RuvBL1/RuvBL2 involved in complex maturation

**Table 2:** His_6_-TEV-MAP/microtubule affinity-regulating kinase 3 (MARK3) (48-370)-HRV3C-AviTag mutants used in this study.

Mutant 1	**I62L, V116I, E139D, T204H, V205Q ^#^**
Mutant 2	I62L, V116I, E139D, T204H, V205Q, **L128I, L130I**
Mutant 3	I62L, V116I, E139D, T204H, V205Q, L128I, L130I, **N66T, F67Y, A68G**
Mutant 4	I62L, V116I, E139D, T204H, V205Q, L128I, L130I, N66T, F67Y, A68G, **F199L**
Mutant 5	I62L, V116I, E139D, T204H, V205Q, L128I, L130I, N66T, F67Y, A68G, F199L, **G137R**
Mutant 6	I62L, V116I, E139D, T204H, V205Q, L128I, L130I, N66T, F67Y, A68G, F199L, G137R, **L72K**

^#^ The mutations indicated in bold refer to the uniqueness of each new construct mutation.

**Table 3:** SPR-determined affinity (K_Dss_) and kinetic (*k_a_*, *k_d_* and K_D_) parameters of the interaction between CypD(43-207) proteins and a reference compound (CYPD-27).

	K_Dss_ ± SD ^¥^ (M)	*k_a_* ± SD (M^−1^·s^−1^)	*k_d_* ± SD (s^−1^)	K_D_ ± SD (M)
Pure CypD(43-207)	2.2 × 10^−7^ ± 1.3 × 10^−8^	9.5 × 10^+5^ ± 3.1 × 10^+5^	2.0 × 10^−1^ ± 6.4 × 10^−2^	2.1 × 10^−7^ ± 1.5 × 10^−8^
In vitro biotinylated CypD(43-207)	1.1 × 10^−7^ ± 8.6 × 10^−9^	4.8 × 10^+5^ ± 2.2 × 10^+5^	9.2 × 10^−2^ ± 8.0 × 10^−3^	1.9 × 10^−7^ ± 1.3 × 10^−7^
Cellular biotinylated CypD(43-207)	2.9 × 10^−7^ ± 8.3 × 10^−9^	3.9 × 10^+5^ ± 3.1 × 10^+4^	1.5 × 10^−1^ ± 4.3 × 10^−3^	3.7 × 10^−7^ ± 1.7 × 10^−8^

^¥^ SD: standard deviation.

**Table 4:** SPR-determined affinity and kinetic parameters of the interaction between YAP proteins and TEAD1(209-426).

	K_Dss_ ± SD (M)	*k_a_* ± SD (M^−1^·s^−1^)	*k_d_* ± SD (s^−1^)	K_D_ ± SD (M)
Biotinylated YAP(61-100)	1.2 × 10^−6^ ± 3.5 × 10^−8^	4.6 × 10^+5^ ± 1.7 × 10^+4^	4.8 × 10^−1^ ± 6.5 × 10^−3^	1.1 × 10^−6^ ± 3.7 × 10^−8^
Cellular biotinylated YAP(2-268)	ND *	1.8 × 10^+6^ ± 1.4 × 10^+6^	8.0 × 10^−2^ ± 2.2 × 10^−2^	4.5 × 10^−8^ ± 1.6 × 10^−8^

* ND: not determined.

**Table 5:** SPR-determined affinity and kinetic parameters of the interaction between the cellular biotinylated YAP(2-268) and TED-347:TEAD1(209-436) and Peptide 17:TEAD1(209-436).

	K_Dss_ ± SD (M)	*k_a_* ± SD (M^−1^·s^−1^)	*k_d_* ± SD (s^−1^)	K_D_ ± SD (M)
TED-347:TEAD1	2.6 × 10^−7^ ± 1.3 × 10^−8^	1.2 × 10^+6^ ± 1.2 × 10^+5^	3.4 × 10^−1^ ± 7.4 × 10^−2^	3.0 × 10^−7^ ± 3.3 × 10^−8^
Peptide 17:TEAD1	>80% Reduction ^¶^	>80% Reduction ^¶^	>80% Reduction ^¶^	>80% Reduction ^¶^

^¶^ As compared with TEAD1 apo-form.

**Table 6:** SPR-determined affinity and kinetic parameters of the interaction between the NUAK2(1-628) and MARK3(48-370) proteins and a known NUAK inhibitor (GSK461364A).

	K_Dss_ ± SD (M)	*k_a_* ± SD (M^−1^·s^−1^)	*k_d_* ± SD (s^−1^)	K_D_ ± SD (M)
NUAK2(1-628)	ND	1.6 × 10^+6^ ± 1.1 × 10^+6^	3.1 × 10^−2^ ± 1.7 × 10^−2^	1.9 × 10^−8^ ± 4.3 × 10^−9^
MARK3(48-370)	1.5 × 10^−5^ ± 1.6 × 10^−6^	ND	ND	ND

**Table 7:** SPR determined kinetic parameters of the interaction between cellular biotinylated MARK3 mutants and GSK461364A.

	*k_a_* ± SD (M^−1^·s^−1^)	*k_d_* ± SD (s^−1^)	K_D_ ± SD (M)
Mutant 2	1.0 × 10^+6^ ± 9.9 × 10^+4^	8.1 × 10^−2^ ± 1.5 × 10^−2^	8.1 × 10^−8^ ± 2.4 × 10^−8^
Mutant 3	8.6 × 10^+5^ ± 2.0 × 10^+5^	2.8 × 10^−2^ ± 3.0 × 10^−3^	3.3 × 10^−8^ ± 4.8 × 10^−9^
Mutant 4	7.6 × 10^+5^ ± 1.2 × 10^+4^	2.9 × 10^−2^ ± 2.8 × 10^−3^	3.8 × 10^−8^ ± 3.4 × 10^−9^
Mutant 5	3.9 × 10^+5^ ± 1.1 × 10^+5^	3.5 × 10^−2^ ± 1.2 × 10^−2^	8.9 × 10^−8^ ± 6.5 × 10^−9^
Mutant 6	4.2 × 10^+5^ ± 4.7 × 10^+4^	2.1 × 10^−2^ ± 1.2 × 10^−3^	5.1 × 10^−8^ ± 1.9 × 10^−9^

**Table 8:** SPR determined kinetic parameters of the interaction between cellular biotinylated DKC1(1-514) and RuvBL1(1-456)/RuvBL2(1-463).

	*k_a_* ± SD (M^−1^·s^−1^)	*k_d_* ± SD (s^−1^)	K_D_ ± SD (M)
RuvBL1(1-456)/RuvBL2(1-463)	6.5 × 10^+5^ ± 1.3 × 10^+5^	1.5 × 10^−2^ ± 1.2 × 10^−3^	2.3 × 10^−8^ ± 2.9 × 10^−9^

## Data Availability

Not available.

## Supplementary Materials

The following supporting information can be downloaded at: https://www.mdpi.com/article/10.3390/bios13100913/s1, Figure S1. Steady-state affinity fit (KDss) for the interaction between pure (A), in vitro biotinylated (B) or cellular biotinylated (C) CypD(43-207) and a known inhibitor (CYPD-27). X-axis: concentration of CYPD-27 (nM); Y-axis: response unit (RU); Figure S2. Steady-state affinity fit (KDss) for the interaction between biotinylated YAP(61-100) and TEAD1(209-436). X-axis: concentration of TEAD1 (nM); Y-axis: response unit (RU); Figure S3. Steady-state affinity fit (KDss) for the interaction between biotinylated YAP(61-100) and TEAD1(209-436) in running buffer supplemented with 30 µM TED-347. X-axis: concentration of TEAD1 (nM); Y-axis: response unit (RU).
