# Supplementary material for: Extract2Chip—Bypassing Protein Purification in Drug Discovery Using Surface Plasmon Resonance
Source: Biosensors (Basel). 2023 Oct 5;13(10):913. doi: 10.3390/bios13100913 (PMC10605449; doi:10.3390/bios13100913)
Supplement: Supplementary file 1 [file biosensors-13-00913-s001.zip › biosensors-2594435-supplementary.pdf]

Supplementary Material

Extract2Chip—Bypassing Protein Purification in Drug Discovery Using Surface Plasmon Resonance

Ana C. F. Paiva [1,2], Ana R. Lemos [1,2], Philipp Busse [1,2], Madalena T. Martins [1,†], Diana O. Silva [1,2], Micael C. Freitas [1,2], Sandra P. Santos [1,2], Filipe Freire [1,2], Evelyne J. Barrey [3,‡], Xavier Manival [4], Lisa Koetzner [3], Timo Heinrich [3], Ansgar Wegener [3], Ulrich Grädler [3], Tiago M. Bandeiras [1,2], Daniel Schwarz [3,*] and Pedro M. F. Sousa [1,2,*]

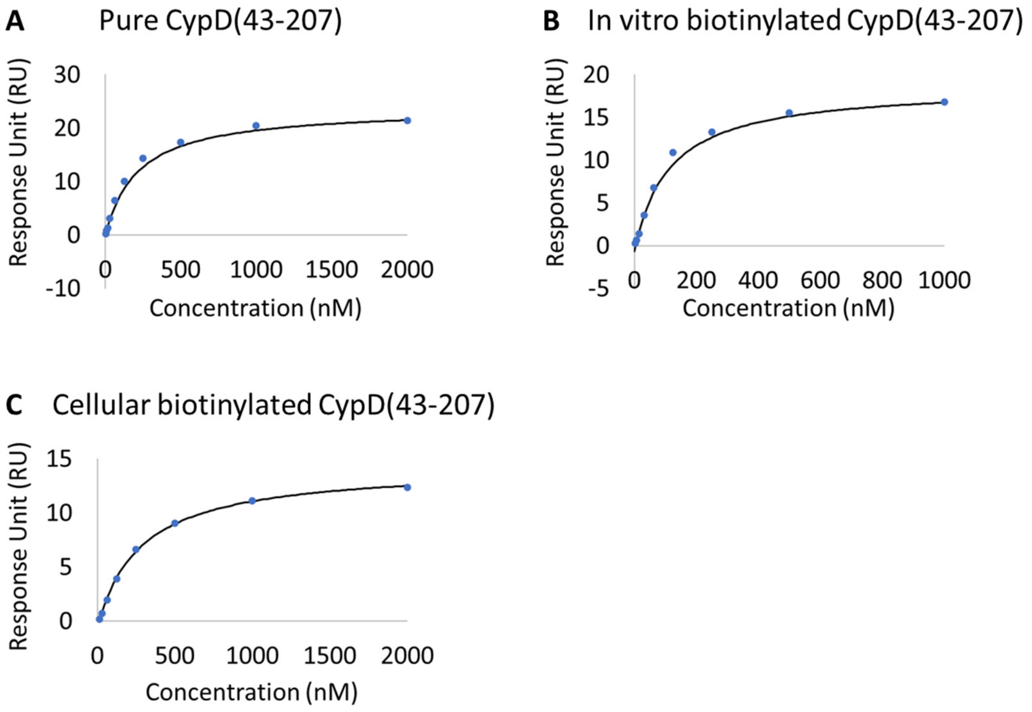

Figure S1. Steady-state affinity fit (KDss) for the interaction between pure (A), in vitro biotinylated (B) or cellular biotinylated (C) CypD(43-207) and a known inhibitor (CYPD-27). X-axis: concentration of CYPD-27 (nM); Y-axis: response unit (RU).

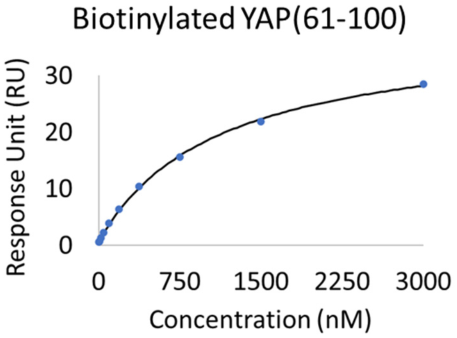

Figure S2. Steady-state affinity fit (KDss) for the interaction between biotinylated YAP(61-100) and TEAD1(209-436). X-axis: concentration of TEAD1 (nM); Y-axis: response unit (RU).

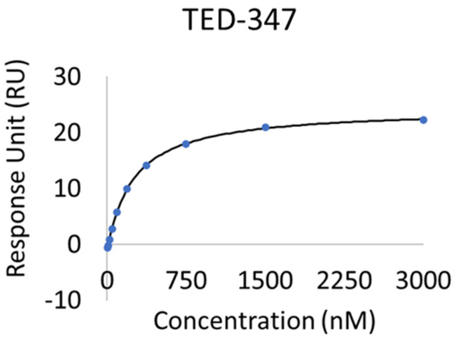

Figure S3. Steady-state affinity fit (KDss) for the interaction between biotinylated YAP(61-100) and TEAD1(209-436) in running buffer supplemented with 30 μM TED-347. X-axis: concentration of TEAD1 (nM); Y-axis: response unit (RU).
